# The impact of diabetes on cognitive decline: potential vascular, metabolic, and psychosocial risk factors

**DOI:** 10.1186/s13195-015-0130-5

**Published:** 2015-06-10

**Authors:** Insa Feinkohl, Jackie F. Price, Mark W.J. Strachan, Brian M. Frier

**Affiliations:** Centre for Population Health Sciences, Medical School, Teviot Place, Edinburgh, EH8 9AG Scotland UK; Metabolic Unit, Western General Hospital, Crewe Road South, Edinburgh, EH4 2XU Scotland UK; The Queen’s Medical Research Institute, University of Edinburgh, College of Medical and Veterinary Medicine, 47 Little France Crescent, Edinburgh, EH16 4TJ Scotland UK

## Abstract

Older people with type 2 diabetes are at increased risk of developing cognitive impairment, for which several potential risk factors have been proposed. The present article reviews evidence in people with type 2 diabetes for associations of cognitive impairment with a range of vascular, metabolic, and psychosocial risk factors, many of which have a higher prevalence in people with type 2 diabetes than in non-diabetic adults of a similar age. Definitive research studies in this field are few in number. The risk factors may be involved in causal pathways or may act as useful markers of cerebrovascular damage (or both), and for which relatively consistent evidence is available, include poor glycemic control, hypoglycemia, microvascular disease, inflammation, and depression. For macrovascular disease, the strength of the association with cognitive impairment appears to depend on which vascular system has been examined. A role for pre-morbid ability in young adulthood as influencing the risk of both diabetes and cognitive impairment has also been suggested. The importance of considering inter-relationships between risk factors when investigating their potential contribution to cognitive impairment in future investigations is discussed.

**Electronic supplementary material:**

The online version of this article (doi:10.1186/s13195-015-0130-5) contains supplementary material, which is available to authorized users.

## Introduction

The global pandemic of diabetes is exerting an ever-increasing burden on health-care systems. The incidence of dementia is also rising worldwide. Diabetes, which is characterized by chronic hyperglycemia, appears to be associated with an increased risk of developing Alzheimer’s disease (AD) and vascular dementia (VaD), both in the general population [1] and in people who have already been diagnosed with a milder form of age-related cognitive impairment (mild cognitive impairment) [2]. With cognitive aging as a continuum, people with type 2 diabetes have been found to experience accelerated cognitive decline within a dementia-free range of between 20 % and 50 % [3], and recent reports have suggested a role of mid-life (rather than late-life) diabetes in particular in promoting this cognitive dysfunction [4, 5].

**Fig. 1:**
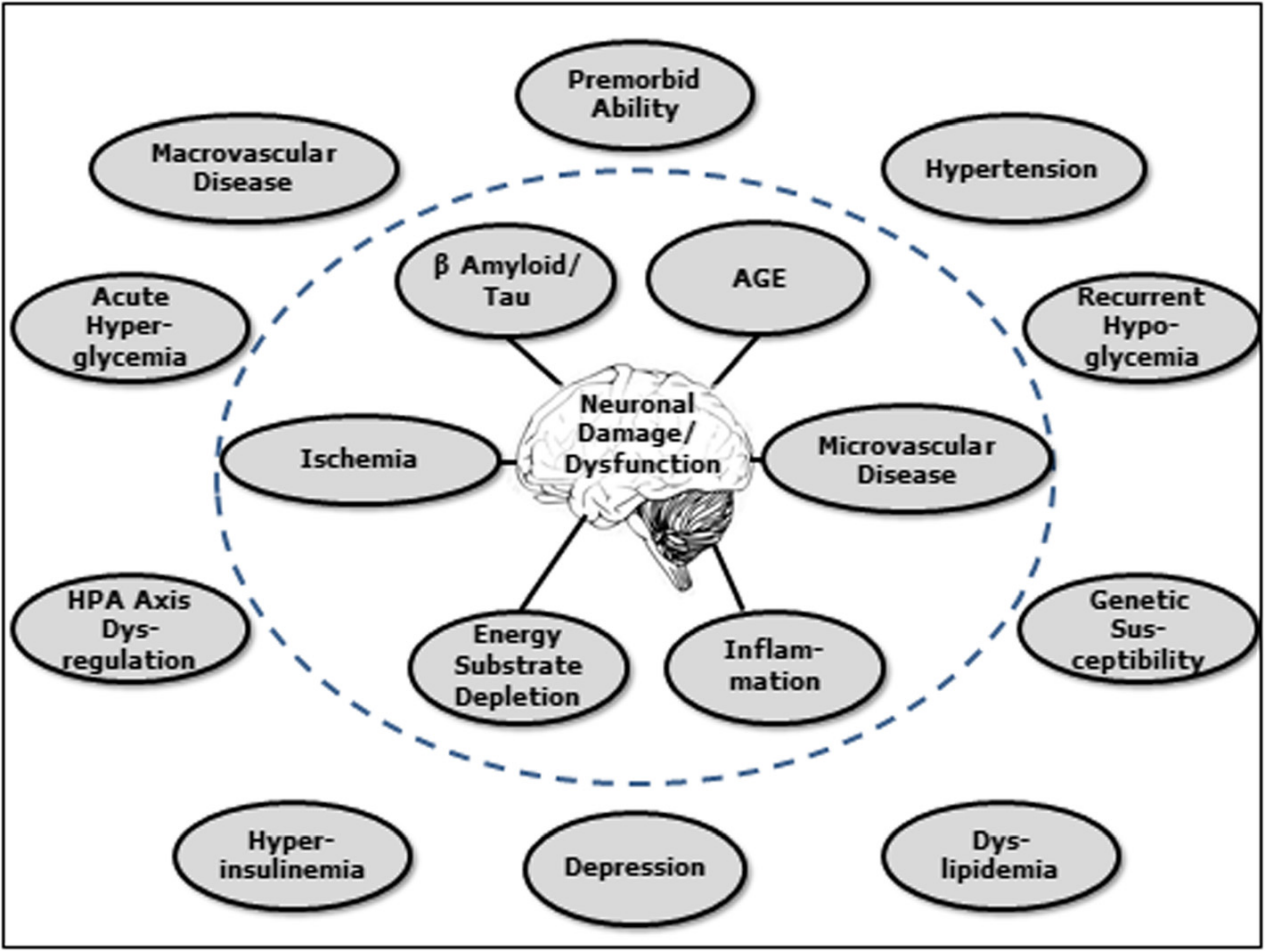
Potential risk factors contributing to the increased risk of cognitive impairment in older adults with type 2 diabetes. AGE, advanced glycation end-products; HPA, hypothalamic-pituitary axis. Adapted from [72]

Numerous vascular, metabolic, and psychosocial factors have a potential role in the development of cognitive impairment in populations with diabetes and may contribute to diabetes-related cognitive decline (Fig. 1). Most factors are inter-related and could influence cognitive ability through a number of different pathophysiological pathways. In this article, we have aimed to provide an overview (rather than a formal systematic review) of the current evidence on risk factors for cognitive impairment in people with diabetes. For that purpose, each risk factor is considered individually and with a focus on prospective epidemiological studies in populations with type 2 diabetes. Where such evidence is lacking, information derived from studies in the general (non-diabetic) population and from adults with type 1 diabetes has been included. It is important to emphasize that even where associations based on observational research are well established, these do not demonstrate causality, and so evaluation of the epidemiological evidence has been supplemented where possible by consideration of intervention studies. Although many of the risk factors are likely to affect cognition through an influence on cerebrovascular disease, on AD-typical pathology or on both, underlying pathophysiological mechanisms are not the main focus of this article, and these have been reviewed extensively elsewhere [6–8]. In addition, possible genetic factors are not addressed. The primary studies identified and reviewed in this article are summarized in Tables 1, 2, 3, 4 and are also provided as supplemental data (Additional file 1).

**Table 1:** Studies of dyslipidemia and cognitive function in type 2 diabetes

Study	Sample	Design	Number	Baseline mean age	Lipids	Cognitive measures	Adjustment variables	Association with cognitive function
Bruce *et al.* [14] (2008)	Patients with type 2 diabetes participating in the Fremantle Diabetes Study; Australia	8-year retrospective, observational	302	Mean 76 ± 5 years	Total cholesterol and HDL at baseline and 8 years earlier	Dementia and MCI identified from screening instruments/clinical interview	Waist-hip ratio	No associations in unadjusted cross-sectional or prospective analyses. Higher total cholesterol 8 years earlier protective of cognitive impairment short of dementia (but not dementia or all cognitive impairment) at baseline (finding independent of waist-hip ratio)
Chen *et al.* [9] (2011)	Patients with type 2 diabetes; China	Cross-sectional, observational	101	Mean 63 ± 8 years	Total cholesterol, LDL, and HDL	MCI identified on the basis of cognitive screening instrument	None	Higher triglycerides, total cholesterol, and LDL in MCI group compared with group free of MCI. Negative correlation of total cholesterol with scores on cognitive screening instrument in patients with MCI. No finding for HDL.
Chen *et al.* [11] (2012)	Patients with type 2 diabetes; China	Cross-sectional, observational	157	Mean 55 ± 7 years	Triglycerides, total cholesterol, LDL, and HDL	MCI identified on the basis of cognitive screening instrument	None	No association
Cukierman-Yaffe *et al.* [13] (2009)	Patients with type 2 diabetes participating in ACCORD-MIND; North America	Cross-sectional analysis of trial on blood pressure, lipids, and glycemic control	2,977	Mean 63 ± 6 years	Hyperlipidemia defined as use of lipid-lowering medication or untreated LDL cholesterol	Digit Symbol Coding (primary outcome), MMSE, Rey Auditory Verbal Learning, and Stroop (secondary outcomes)	Age	Association of hyperlipidemia with better performance on Digit Symbol Coding. No findings for other cognitive tests.
Perlmutter *et al.* [10] (1988)	Patients with type 2 diabetes; USA	Cross-sectional, observational	246	Range 55-74 years	Triglycerides and total cholesterol	Digit Symbol Coding, Digit Span, and simple reaction time	Plasma glucose, HbA1c, body mass index, and history of hypertension	Lower cognitive function in ‘high’ compared with ‘low’ triglyceride groups (fully adjusted analysis); association of cholesterol with triglyceride (unadjusted analysis).
Umegaki *et al.* [16] (2014)	Patients with type 2 diabetes; Japan	Six-year prospective, observational	79	Mean 74 ± 5 years	Mean of HDL and LDL measured at baseline and annual follow-ups	Composite score from MMSE, Digit Symbol Coding, Stroop, and word recall. Analyses of ‘decliners’ versus ‘non-decliners’ on the basis of composite score and individual cognitive tests.	Age, education, estimated glomerular filtration rate, renin-angiotensin system inhibitor use, paraventricular hyperintensities, and deep white matter hyperintensities	Lower mean 6-year HDL in ‘decliners’ compared with ‘non-decliners’ on composite score and Stroop (unadjusted analysis). No finding for LDL. Mean 6-year HDL significant predictor in model of risk of decline on composite score (fully adjusted analysis).
Williamson *et al.* [17] (2014)	Patients with type 2 diabetes participating in ACCORD-MIND lipid arm, receiving simvastatin + fenofibrate or simvastatin + placebo; North America	40-month trial on blood pressure, lipids, and glycemic control	1,538	Mean 62 ± 6 years	Successful manipulation of cholesterol levels (groups differed on cholesterol following intervention).	Total brain volume at baseline and 40 months, Digit Symbol Coding (primary outcome), MMSE, Rey Auditory Verbal Learning, and Stroop (secondary outcomes) at baseline and 20 and 40 months	Glycemia treatment arm, visit effect, clinical center, and history of cardiovascular disease	No difference in 20- or 40-month cognitive decline or 40-month change in total brain volume between intervention and control groups of lipid trial
Van Harten *et al.* [12] (2007)	Patients with type 2 diabetes; The Netherlands	Cross-sectional, observational	92	Mean 73 ± 6 years	Total cholesterol/HDL ratio	Cognitive screening instruments, composite scores of four cognitive domains derived from scores on battery of 10 cognitive tests	Duration of diabetes, HbA1c, insulin use, hypertension, and polyneuropathy	No association
Yanagawa *et al.* [15] (2011)	Patients with diabetes receiving exercise program four times per week versus none; Japan	12-week trial on physical exercise intervention	16	Mean 71 ± 4 years	HDL and LDL (no intervention effect on HDL and LDL)	MMSE, word recall, Digit Symbol Coding, Stroop, and Trail-Making Test	Age, education, and body mass index	No difference in cognitive function between treatment groups following intervention

*ACCORD-MIND*, Action to Control Cardiovascular Risk in Diabetes-Memory in Diabetes; *HDL*, high-density lipoprotein; *LDL*, low-density lipoprotein; *MCI*, mild cognitive impairment; *MMSE*, Mini-Mental State Examination

**Table 2:** Studies of hypertension and cognitive function in type 2 diabetes

Study	Sample	Design	Number	Baseline mean age	Blood pressure	Cognitive measures	Adjustment variables	Association with cognitive function
Chen *et al.* [11] (2012)	Patients with type 2 diabetes; China	Cross-sectional, observational	157	Mean 55 ± 7 years	Hypertension defined on the basis of systolic blood pressure and diastolic blood pressure	MCI identified on the basis of cognitive screening instrument	None	Higher prevalence of hypertension in group with MCI compared with group free of MCI. Negative correlation of presence with hypertension with cognitive scores. No findings for blood pressure as continuous measure.
Bruce *et al.* [14] (2008)	Patients with type 2 diabetes participating in the Fremantle Diabetes Study; Australia	8-year retrospective, observational	302	Mean 76 ± 5 years	Systolic blood pressure and diastolic blood pressure at baseline and 8 years earlier	Dementia and MCI identified from screening instruments/clinical interview	Age and duration of diabetes	Prospective analyses: higher diastolic blood pressure 8 years earlier associated with increased risk of AD (but not MCI or any dementia) at follow-up. No findings in cross-sectional analyses.
Bruce *et al.* [21] (2008)	Patients with type 2 diabetes participating in the Fremantle Diabetes Study; Australia	8-year retrospective, 2-year prospective, observational	205	Mean 75 ± 4 years	Systolic blood pressure and diastolic blood pressure measured 8 years prior to baseline cognitive assessment	Dementia and MCI identified from screening instruments/clinical interview at baseline and 2-year follow-up. ‘Cognitive decline’ defined as downward conversion between ‘normal’, MCI, and dementia.	None	No association
Cukierman-Yaffe *et al.* [13] (2009)	Patients with type 2 diabetes participating in ACCORD-MIND; North America	Cross-sectional analysis of trial on blood pressure, lipids, and glycemic control	2,977	Mean 63 ± 6 years	Hypertension defined as use of anti-hypertensive medication or self-report of hypertension	Digit Symbol Coding (primary outcome), MMSE, Rey Auditory Verbal Learning, and Stroop (secondary outcomes)	Age	Association of hypertension with poorer performance on Digit Symbol Coding. No findings for other cognitive tests.
Hassing *et al.* [19] (2004)	OCTO-Twin Study of people without diabetes/hypertension, diabetes or hypertension alone, or co-morbid diabetes/hypertension; Sweden	6-year prospective, observational	258	Mean 83 ± 2 years	Hypertension defined as use of anti-hypertensive medication or on the basis of systolic blood pressure and diastolic blood pressure from medical records	MMSE administered at baseline and at 2-year intervals, dementia diagnosis prevalent at baseline, and incident dementia diagnosis	Age, sex, education, smoking, angina, MI, CHF, stroke, and TIA	Co-morbid diabetes/hypertension associated with steeper decline on MMSE (compared with group free of both conditions).
								Statistically non-significant trend for higher prevalence and incidence of dementia in co-morbid diabetes/hypertension group than in remaining groups.
Johnson *et al.* [22] (2012)	National cohort of veterans with diabetes; USA	2-year retrospective, observational study of hospital records	377,838	Mean 76 ± 6 years	ICD codes for hypertension at baseline (2 years before analysis of incident dementia)	ICD codes for incident dementia diagnosis	Age, ethnicity, geographic area, duration of diabetes, co-morbidity according to HCC scores, and medication use	8 % increased risk of developing dementia during follow-up in patients with co-morbid hypertension at baseline.
								Decreased risk in patients on anti-hypertensive medication (effect size dependent on medication), except for increased risk in patients receiving α-adrenoceptor blockers.
Manschot *et al.* [20] (2006)	Patients with type 2 diabetes participating in the Utrecht Diabetic Encephalopathy Study; The Netherlands	Cross-sectional, observational	122	Mean 66 ± 6 years	Hypertension defined on the basis of systolic blood pressure and diastolic blood pressure or use of anti-hypertensive medication	Composite scores on five cognitive domains from 11 cognitive tests, estimate of pre-morbid ability, cortical atrophy, and white matter lesions	Age, sex, and estimated pre-morbid ability	Statistically non-significant trend for lower scores on all cognitive domains except memory in patients with hypertension. Higher blood pressure associated with higher scores on memory domain and with greater severity of white matter lesions.
Manschot *et al.* [25] (2007)	Patients with type 2 diabetes participating in the Utrecht Diabetic Encephalopathy Study; The Netherlands	Cross-sectional, observational	122	Mean 66 ± 6 years	Hypertension defined on the basis of systolic blood pressure and diastolic blood pressure or use of anti-hypertensive medication	Composite score from 11 cognitive tests, estimate of pre-morbid ability, cortical atrophy, and white matter lesions	Age, sex, and estimated pre-morbid ability	Statistically non-significant trend for lower cognitive function in patients with hypertension (reaches statistical significance in final model including age, estimated pre-morbid ability, lipid-lowering drugs, and history of any vascular event). Higher blood pressure associated with greater severity of white matter lesions.
Umegaki *et al.* [16] (2014)	Patients with type 2 diabetes; Japan	6-year prospective, observational	79	Mean 74 ± 5 years	Mean of systolic blood pressure and diastolic blood pressure measured at baseline and annual follow-ups	Composite score from MMSE, Digit Symbol Coding, Stroop, and word recall. Analyses of ‘decliners’ versus ‘non-decliners’ on bases of composite score and individual cognitive tests.	None	No association
Williamson *et al.* [17] (2014)	Patients with type 2 diabetes participating in ACCORD-MIND blood pressure arm, with systolic blood pressure goal of 120 versus 140 mmHg; North America	40-month trial on blood pressure, lipids, and glycemic control	1,439	Mean 62 ± 6 years	Successful manipulation of blood pressure (groups differed on blood pressure following intervention)	Total brain volume at baseline and 40 months, Digit Symbol Coding (primary outcome), MMSE, Rey Auditory Verbal Learning, and Stroop (secondary outcomes) at baseline and 20 and 40 months	Glycemia treatment arm, visit effect, clinical center, and history of cardiovascular disease	No difference in 20- or 40-month cognitive decline treatment and control groups. Greater reduction in total brain volume in intervention than in control group.

*ACCORD-MIND*, Action to Control Cardiovascular Risk in Diabetes-Memory in Diabetes; *AD*, Alzheimer’s dementia; *CHF*, congestive heart failure; *HCC*, Centers for Medicare and Medicaid Services’ Hierarchical Condition Categories; *ICD*, International Classification of Diseases; *MCI*, mild cognitive impairment; *MI*, myocardial infarction; *MMSE*, Mini-Mental State Examination; OCTO-Twin Study, Origin of Variance in the Old Old Study: Octogenarian Twins; TIA, transient ischemic attack

**Table 3:** Studies of hyperglycemia or hyperinsulinemia and cognitive function in type 2 diabetes

Study	Sample	Design	Number	Baseline mean age	Hyperglycemia/ hyperinsulinemia	Cognitive measures	Adjustment variables	Association with cognitive function
Abbatecola *et al.* [30] (2006)	Patients with diabetes free of vascular disease, receiving repaglinide or glibenclamide; Italy	12-month trial on glycemic control	156	Mean 74 ± 2 years	Variation in post-prandial blood glucose, fasting plasma glucose, and HbA1c. (No difference between groups in reduction of HbA1c and plasma glucose during trial. Decline in variation in post-prandial glucose only in group treated with repaglinide.)	Composite score of attention/executive function (Trail-Making Test, Digit Span, and verbal fluency), MMSE, cortical atrophy, and white matter lesions	Age, education, physical activity, depression, blood pressure, cIMT, insulin resistance, and body mass index	Association of higher variation in fasting plasma glucose and post-prandial blood glucose with lower cognitive function across groups at baseline (fully adjusted analyses). Composite score and MMSE declined in glibenclamide but not in repaglinide group during trial (analyses controlling only for HbA1c and variation in fasting plasma glucose).
Bruce *et al.* [14] (2008)	Patients with type 2 diabetes participating in the Fremantle Diabetes Study; Australia	8-year retrospective, observational	302	Mean 76 ± 5 years	HbA1c at baseline and 8 years earlier	Dementia and MCI identified from screening instruments/clinical interview	None	No association
Bruce *et al.* [21] (2008)	Patients with type 2 diabetes participating in the Fremantle Diabetes Study; Australia	8-year retrospective, 2-year prospective, observational.	205	Mean 75 ± 4 years	HbA1c 8 years prior to baseline cognitive assessment	Dementia and MCI identified from screening instruments/clinical interview at baseline and at 2-year follow-up. ‘Cognitive decline’ defined as downward conversion between ‘normal’, MCI, and dementia.	None	No association
Chen *et al.* [9] (2011)	Patients with type 2 diabetes; China	Cross-sectional, observational	101	Mean 63 ± 8 years	HbA1c	MCI identified on the basis of cognitive screening instrument	None	Higher HbA1c in group with MCI compared with group free of MCI
Chen *et al.* [11] (2012)	Patients with type 2 diabetes; China	Cross-sectional, observational	157	Mean 55 ± 7 years	HbA1c	MCI identified on the basis of cognitive screening instrument	None	No association
Cukierman-Yaffe *et al.* [13] (2009)	Patients with type 2 diabetes participating in ACCORD-MIND; North America	Cross-sectional analysis of trial on blood pressure, lipids, and glycemic control	2,977	Mean 63 ± 6 years	HbA1c and fasting plasma glucose	Digit Symbol Coding (primary outcome), MMSE, Rey Auditory Verbal Learning, and Stroop (secondary outcomes)	Total of 18 demographic and clinical risk factors	Higher HbA1c associated with lower Digit Symbol Coding. Findings for other cognitive tests did not survive full adjustment. No findings for fasting plasma glucose.
Launer *et al.* [28] (2011)	Patients with type 2 diabetes participating in ACCORD-MIND trial, with HbA1c targets of <6.0 % versus 7.0 % to 7.9 %; North America	40-month trial on blood pressure, lipids and glycemic control	2,977	Mean 62 ± 6 years	Successful manipulation of glycemic control. (Treatment groups differed in glycemic control following intervention.)	Total brain volume and abnormal white matter at baseline and 40 months. Digit Symbol Coding (primary outcome), MMSE, Rey Auditory Verbal Learning, and Stroop (secondary outcomes) at baseline and 20 and 40 months.	Second trial assignment (lipid or blood pressure trials), group allocation within second trial assignment, clinical center, and history of cardiovascular disease	No difference in 20- or 40-month cognitive decline between intervention groups (fully adjusted analyses). Total brain volume declined at slower rate in intensively treated compared with standard treatment groups (independent of adjustment variables and of age, sex, duration of diabetes, Digit Symbol Coding). Greater abnormal white matter in intensively treated compared with standard treatment group at 40 months.
Manschot *et al.* [20] (2006)	Patients with type 2 diabetes participating in the Utrecht Diabetic Encephalopathy Study; The Netherlands	Cross-sectional, observational	122	Mean 66 ± 6 years	HbA1c	Composite scores on five cognitive domains from 11 cognitive tests, estimate of pre-morbid ability, cortical atrophy, and white matter lesions	Age, sex, and estimated pre-morbid ability	Association of higher HbA1c with steeper estimated lifetime decline in processing speed. No association of HbA1c with brain imaging data.
Manschot *et al.* [25] (2007)	Patients with type 2 diabetes participating in the Utrecht Diabetic Encephalopathy Study; The Netherlands	Cross-sectional, observational	122	Mean 66 ± 6 years	HbA1c and plasma insulin	Composite score from 11 cognitive tests, estimate of pre-morbid ability, cortical atrophy, and white matter lesions	Age, sex, and estimated pre-morbid ability	Association of higher HbA1c with steeper estimated lifetime decline in overall cognitive function. Association of higher insulin with greater severity of white matter lesions.
Ravona-Springer *et al.* [27] (2014)	Patients with type 2 diabetes participating in the Israel Diabetes and Cognitive Decline Study; Israel	12-year retrospective observational	835	Mean 73 ± 5 years	Data on HbA1c from diabetes register (mean 18 ± 10 measurements per patient). Six HbA1c trajectories identified (for example, high/increasing and high/stable).	Measured at 12 years only: CDR, MMSE, and battery of seven cognitive tests. Sum of z-scores calculated for four cognitive domains.	Age, sex, education, cardiovascular disease, years in diabetes register, anti-diabetes treatment, and depression	Associations of HbA1c trajectories with level of overall cognitive function, semantic categorization, and executive function. Relatively poorest cognitive function in high/decreasing group and high/increasing groups. Relatively highest performance in low/stable group.
Ryan *et al.* [29] (2006)	Patients with type 2 diabetes, receiving rosiglitazone or glibenclamide; USA	24-week trial	145	Mean 60 ± 1 years	Successful manipulation of glycemic control and insulin sensitivity. (Treatment groups differed in fasting plasma glucose and fasting serum insulin following intervention.)	CANTAB, Digit Symbol Coding, Rey Auditory Verbal Learning, and estimate of pre-morbid ability	Age, center, pre-morbid ability, and baseline measurement of fasting plasma glucose/insulin	Cognitive function improved equally in both treatment groups (fully adjusted analysis). Correlation of reduction in fasting plasma glucose with improvements in working memory across groups (unadjusted analysis). No finding for insulin.
Seaquist *et al.* [41] (2013)	Patients with type 2 diabetes participating in ACCORD-MIND, with HbA1c targets of less than 6.0 % versus 7.0 % to 7.9 %; North America	40-month trial on blood pressure, lipids, and glycemic control	2,977	Mean 62 ± 6 years	Treatment with insulin at enrolment and during trial	Digit Symbol Coding at baseline and 20 and 40 months	Total of 21 demographic, lifestyle, and clinical covariates	Association of insulin use at enrolment with lower baseline cognitive function. Loss of statistical significance upon full adjustment. No association of insulin use during trial with 40-month cognitive decline in standard treatment group. Association of insulin use with steeper 40-month cognitive decline in intensive treatment group; loss of statistical significance upon full adjustment.
Umegaki *et al.* [16] (2014)	Patients with type 2 diabetes; Japan	6-year prospective, observational	79	Mean 74 ± 5 years	Mean of HbA1c and plasma immunoreactive insulin at baseline and annual follow-ups.	Composite score from MMSE, Digit Symbol Coding, Stroop, and word recall. Analyses of ‘decliners’ versus ‘non-decliners’ on bases of composite score and individual cognitive tests.	None	No associations for HbA1c or insulin, except for higher mean 6-year insulin in ‘decliners’ compared with ‘non-decliners’ on Stroop.
Yanagawa *et al.* [15] (2011)	Patients with diabetes receiving exercise program four times/week versus none; Japan	12-week trial on physical exercise intervention	16	Mean 71 ± 4 years	HbA1c, fasting blood glucose, GIR, MCR in euglycemic clamp, and immunoreactive insulin. (No intervention effect on any of these measurements.)	MMSE, word recall, Digit Symbol Coding, Stroop, and Trail-Making Test	Age, education, and body mass index	No difference in cognitive function between groups following intervention. Across groups, changes in HbA1c and changes in GIR correlated with changes in word recall. Changes in fasting blood glucose correlated with changes in Trail-Making.

*ACCORD-MIND*, Action to Control Cardiovascular Risk in Diabetes-Memory in Diabetes; *CANTAB*, Cambridge Neuropsychological Test Automated Battery; *CDR*, Clinical Dementia Rating Scale; *cIMT*, carotid intima-media thickness; *GIR*, glucose infusion rate; *MCI*, mild cognitive impairment; *MCR*, metabolic clearance rate; *MMSE*, Mini-Mental State Examination

## Vascular and metabolic risk factors

### Dyslipidemia

Although dyslipidemia is common in type 2 diabetes, few observational studies have examined whether an association exists between plasma lipid concentrations and cognitive function (Table 1). Cognitive function has been reported to be significantly poorer in people with type 2 diabetes who have elevated levels of plasma triglycerides [9, 10] and in those with higher cholesterol levels [9], but neither of these observations has been confirmed [11, 12]. Two investigations even reported protective effects: in one cross-sectional study, dislipidemia was associated with better performance on a task of processing speed [13], and higher total cholesterol was found to decrease the risk of subsequent cognitive impairment short of dementia during an 8-year period in the Fremantle Diabetes Study [14]. However, a small intervention study on the effects of physical exercise on insulin resistance over a period of 12 weeks (which was unsuccessful in inducing a change in insulin resistance) [15] and two further prospective observational studies [16, 17] failed to find any association between plasma lipid profiles and subsequent cognitive decline or risk of impairment, with the exception of an apparent association between lower mean high-density lipoprotein during a 6-year period and a steeper-than-expected cognitive decline in a small Japanese study during the same time period [16].

In the Action to Control Cardiovascular Risk in Diabetes-Memory in Diabetes (ACCORD-MIND) randomized controlled trial (RCT), almost 3,000 older people with type 2 diabetes were assigned either to intensive treatment of hyperglycemia or to standard therapy [17]. Around 50 % of participants also entered the only RCT to date to address the effects of a reduction in plasma lipid levels on cognitive decline in people with type 2 diabetes (the other 50 % participated in a trial of anti-hypertensives). Despite a greater reduction in cholesterol levels in patients who received fenofibrate plus simvastatin compared with those receiving placebo plus simvastatin, cognitive function in the two groups declined at similar rates during a 40-month follow-up period [17]. A review of RCTs performed in the general (predominantly non-diabetic) population also concluded that reducing plasma cholesterol does not influence late-life cognitive function [18], consistent with findings from observational studies performed in the general population [2]. The role of dyslipidemia in the development of cognitive impairment in people with diabetes is therefore uncertain.

### Hypertension

Hypertension is common in people with type 2 diabetes and, in general, has received more attention than dyslipidemia as a potential risk factor for diabetes-related cognitive impairment (Table 2). Cross-sectional studies have revealed trends for increased prevalence of hypertension in patients with lower cognitive function [11, 13, 19, 20], but cross-sectional analyses of blood pressure as a continuous measure have failed to identify similar associations [11, 14, 21]. On the other hand, some [14, 19, 22], though not all [17, 21], prospective studies have found a relationship between baseline blood pressure or hypertension and the subsequent risk of cognitive decline. In the Fremantle Diabetes Study, higher baseline diastolic blood pressure was associated with an increased risk of incident AD after 8 years [14], and in an investigation of people over 80 years of age, the coexistence of hypertension appeared to exacerbate diabetes-related cognitive decline during a 6-year follow-up and to increase the risk of dementia [19]. Similarly, a retrospective study that examined the hospital records of almost 380,000 older patients with diabetes showed that co-morbid hypertension increased the 2-year risk of dementia; treatment with anti-hypertensive medication (other than α-adrenoceptor blockers, with which the risk of dementia was increased) further diminished the risk of dementia by between 4 % and 24 % depending on the precise type of drug used [22]. In contrast with these findings, the blood pressure trial of the ACCORD-MIND study did not demonstrate a difference in cognitive decline over a period of 40 months between a group of patients who received intensive anti-hypertensive therapy and a group on conventional treatment, despite the success of the trial in producing a difference in blood pressure between the two treatment groups [17]. However, a direct association between blood pressure and cognitive decline was not explored. In the general (non-diabetic) population, the results of observational studies and of RCTs investigating links between hypertension and cognitive impairment have also, in the main, been negative [2, 23]. Therefore, although hypertension causes cerebrovascular disease and, as such, represents a good candidate for a cognitive risk factor, its role in the development of cognitive decline during aging in either the diabetic or non-diabetic population remains unproven.

### Hyperglycemia

Raised blood glucose levels within the non-diabetic or pre-diabetic range have consistently been associated with cognitive impairment, with the strength of the association increasing with advancing age [24]. Given that diabetes is characterized by persistently raised blood glucose levels, a causative role for hyperglycemia in diabetes-associated cognitive decline would seem likely. However, the findings from cross-sectional analyses on the association of HbA1c with cognitive function [9, 11, 13, 14, 20, 25] and cognitive decline [14, 16, 21] in people with type 2 diabetes have been inconsistent (Table 3), potentially due to the different ages of the study populations. Overall, the association of type 2 diabetes with increased cognitive impairment appears to be relatively weak before the age of 70 years, provided that good glycemic control is maintained, and it is only in older patients that cognitive decrements related to chronic hyperglycemia become apparent [26]. More recently, a retrospective analysis of a cohort of people with type 2 diabetes, in whom 12-year data on HbA1c were available from a diabetes register, showed that in addition to increments in blood glucose levels over time, poor glycemic control long-term predicted a lower level of late-life cognitive function, despite a trend toward improved glycemic control by intensifying therapy [27]. This is consistent with the evidence showing damaging effects of mid-life diabetes on the risk of late-life cognitive impairment [4, 5] and suggests that irreversible damage may already have occurred to predispose people to cognitive impairment by the time that aggressive glucose-lowering treatment was commenced.

In one of a number of intervention studies, changes in blood glucose levels due to physical exercise correlated with changes in cognitive function [15]. The ACCORD-MIND study also found a statistically non-significant trend for decelerated decline in processing speed at 20 months in the group with intensive therapy for glycemic control (who achieved relatively greater glycemic control) as compared with the conventional treatment group (with resulting poorer glycemic control), although this difference was no longer apparent at 40 months [28]. Two smaller trials of patients with type 2 diabetes have reported significant associations between improved glycemic control and cognitive function. In one, improvements in glycemic control in both treatment groups due to treatment with either rosiglitazone or glibenclamide (glyburide) correlated with improvement in working memory over a period of 24 weeks [29]. In another, a reduction in post-prandial glucose excursions with repaglinide was associated with a decline in cognitive function over a period of 12 months compared with subjects who received glibenclamide and did not show such a change in glucose excursions; the decline in HbA1c was of similar magnitude in the two treatment groups, suggesting a specific role for post-prandial glucose excursions [30]. Whereas overall a recent systematic review combining the evidence from observational studies and from RCTs concluded that both hyperglycemia and glucose excursions are weakly associated with poorer cognitive function in people with type 2 diabetes [31], a meta-analysis restricted to RCTs suggested that improvement in glycemic control was unrelated to cognitive decline [32], illustrating the need for further evaluation of hyperglycemia as a potentially modifiable cognitive risk factor.

### Hypoglycemia

Few studies have investigated the effect of previous exposure to recurrent hypoglycemia on cognitive function in people with type 2 diabetes. Heterogeneity with respect to how ‘hypoglycemia’ has been defined presents a major problem for interpretation of results, with recorded events ranging from asymptomatic biochemical hypoglycemia to severe disabling hypoglycemia (Table 4).

**Table 4:** Studies of hypoglycemia and cognitive function in type 2 diabetes

Study	Sample	Design	Number	Baseline mean age	Hypoglycemia	Cognitive measures	Adjustment variables	Association with cognitive function
Aung *et al.* [33] (2012)	Patients with type 2 diabetes participating in the Edinburgh Type 2 Diabetes Study; Scotland	Cross-sectional, observational	1,066	Mean 68 ± 4 years	Baseline self-report of history of SH (defined as episode requiring assistance)	MMSE, composite score from seven cognitive tests, and estimate of pre-morbid ability	Age, sex, duration of diabetes, anti-diabetes medication, depression, alcohol, smoking, blood pressure, HbA1c, stroke, TIA, MI, angina, and retinopathy. Analyses of estimated lifetime decline additionally adjusted for estimated pre-morbid ability.	History of SH associated with lower cognitive function and steeper estimated lifetime decline (fully adjusted analyses). Linear negative relationship between number of episodes of SH in the year before cognitive testing and cognitive function (analysis controlling for age and sex).
Bruce *et al.* [14] (2008)	Patients with type 2 diabetes participating in the Fremantle Diabetes Study; Australia	8-year retrospective, observational	302	Mean 76 ± 5 years	Hypoglycemia resulting in coma or hospitalization, self-reported at baseline and 8 years earlier	Dementia and MCI identified from screening instruments/clinical interview	None	Cross-sectional analysis: increased prevalence of history of hypoglycemia in groups with poorer cognitive function. No findings in prospective analyses.
Bruce *et al.* [21] (2008)	Patients with type 2 diabetes participating in the Fremantle Diabetes Study; Australia	8-year retrospective, 2-year prospective, observational	205	Mean 75 ± 4 years	Hypoglycemia resulting in coma or hospitalization, self-reported 8 years prior to baseline cognitive assessment	Dementia and MCI identified from screening instruments/clinical interview at baseline and at 2-year follow-up. ‘Cognitive decline’ defined as downward conversion between ‘normal’, MCI, and dementia.	None	No association
Bruce *et al.* [34] (2009)	Patients with type 2 diabetes participating in the Fremantle Diabetes Study; Australia	5-year prospective, observational	302	Mean 76 ± 5 years	1. Self-reported medical assistance or unconsciousness or both.	Dementia and MCI identified from screening instruments/clinical interview. ‘All cognitive impairment’ summarizes groups of dementia and MCI.	Based on preliminary associations of covariates with cognition, the following adjustment variables were selected for: analyses of MCI: age, sex, education, and cardiovascular disease; for analyses of dementia: duration of diabetes and peripheral arterial disease; for analyses of all cognitive impairment: cardiovascular disease, peripheral arterial disease, and duration of diabetes.	Cross-sectional analyses: presence of MCI and all cognitive impairment associated with history of all three measures of hypoglycemia (fully adjusted analyses). No finding for dementia. Prospective analyses: no association of baseline history of hypoglycemia and risk of ‘cognitive decline’ in patients free of cognitive impairment at baseline (unadjusted). Increased risk of first-ever incident HSH in group with dementia at baseline (adjusted for insulin use, body mass index, inability to self-manage medication, and history of severe hypoglycemia). No finding on risk of subsequent hypoglycemia in patients with MCI at baseline.
					2. Episodes rated by medical staff as ‘doctor-verified’. 3. Codes for ambulance or emergency treatment for hypoglycemia in hospital records (HSH).	‘Cognitive decline’ defined as conversion between unimpaired, MCI, and dementia.		
de Galan *et al.* [35] (2009)	Patients with type 2 diabetes participating in ADVANCE arm on glycemic control, receiving standard target versus target HbA1c ≤6.5 %; Australia	5-year trial on effects of intensified blood pressure control and intensified glycemic control	11,140	Mean 66 ± 6 years	Incident SH defined as blood glucose <2.8 mmol/L or symptoms consistent with hypoglycemia with absence of another cause and requiring external assistance. Incident mild hypoglycemia defined as self-treated episode.	At baseline and 2-year intervals: MMSE followed by clinical interview for patients with MMSE <24 or suspected dementia. ‘Normal’ cognitive function defined as MMSE ≥28; ‘mild dysfunction’ as MMSE = 24-27; ‘severe dysfunction’ as MMSE <24. Additional use of MMSE as continuous measure.	Age, sex, treatment arm, education, duration of diabetes, blood pressure, hypertension, HbA1c, cholesterol, body mass index, macrovascular disease, microvascular disease, smoking, and alcohol	Prospective analyses (unadjusted): increased risk of SH (but not any hypoglycemia) in groups with ‘mild dysfunction’ and ‘severe dysfunction’ (versus ‘normal’ group). For ‘severe dysfunction’, but not ‘mild dysfunction’, finding survived full adjustment. Each unit-lower baseline MMSE score associated with 10 % increased risk of SH (adjusted for age, sex, education, and treatment group). Increased risk of hypoglycemia in treatment group with intensified glycemic control, but finding similar across cognitive groups. No difference in cognitive decline between treatment groups.
Feinkohl *et al.* [38] (2014)	Patients with type 2 diabetes participating in the Edinburgh Type 2 Diabetes Study; Scotland	4-year prospective, observational	1,066	Mean 68 ± 4 years	Self-reported history of episode requiring assistance at baseline (prevalent SH) and during follow-up (incident SH)	MMSE, composite score from seven cognitive tests, and estimate of pre-morbid ability	Age, sex, cholesterol, blood pressure, smoking, HbA1c, TIA, stroke, MI, and angina	History of SH and incident SH both associated with lower cognitive function at year 4 and with increased rate of 4-year cognitive decline. Incident SH associated with steeper estimated lifetime decline. Baseline lower cognitive function predicted increased risk of incident SH.
Launer *et al.* [28] (2011)	Patients with type 2 diabetes participating in ACCORD and ACCORD-MIND, with HbA1c targets of less than 6.0 % versus 7.0 % to 7.9 %; North America	40-month trial on blood pressure, lipids, and glycemic control	2,977	Mean 62 ± 6 years (ACCORD-MIND)	Increased risk of episode of hypoglycemia requiring medical assistance and of episode of hypoglycemia requiring any assistance in treatment arm with intensified glycemic control in ACCORD (n = 10,251)	Total brain volume at baseline and 40 months. Digit Symbol Coding (primary outcome), MMSE, Rey Auditory Verbal Learning, and Stroop (secondary outcomes) at baseline and 20 and 40 months.	Second trial assignment (lipid or blood pressure trials), group allocation within second trial assignment, clinical center, and history of cardiovascular disease	No difference in 20- or 40-month cognitive decline between treatment groups in ACCORD-MIND substudy of ACCORD (fully adjusted analyses). Total brain volume declined at slower rate in intensively treated compared with standard treatment group (independent of adjustment variables, and of age, sex, duration of diabetes, and Digit Symbol Coding score). Greater abnormal white matter in intensively treated compared with standard treatment group at 40 months.
Lin and Sheu [40] (2013)	Patients with diabetes (>45 years); Taiwan	3-year retrospective ascertainment of hypoglycemia. Dementia ascertained in subsequent 4 years	15,000	Mean 64 ± 10 years	ICD codes for any hypoglycemia from inpatient and outpatient medical records	ICD codes for dementia from inpatient and outpatient medical records	Age, sex, insulin use, cardiovascular disease, hypertension, ischemic heart disease, chronic kidney disease, and cholesterol	Hypoglycemia associated with increased risk of subsequent dementia diagnosis. Linear relationship of number of episodes with dementia risk.
Punthakee *et al.* [36] (2012)	Patients with type 2 diabetes participating in ACCORD-MIND, with HbA1c targets of <6.0 % versus 7.0 % to 7.9 %; North America	40-month trial on blood pressure, lipids and glycemic control	2,956	Mean 62 ± 6 years	1. SH defined as self-reported <2.8 mmol/L or symptoms that resolved with use of glucose or similar. 2. HMA defined as episode requiring medical assistance (hospitalization; care in emergency department/ by emergency personnel). 3. HAA defined as episode requiring any assistance.	Digit Symbol Coding (primary outcome), MMSE, Rey Auditory Verbal Learning, and Stroop (secondary outcomes) at baseline and 20 and 40 months	Age, education, language of test administration, depression, second trial assignment (lipid or blood pressure trials), group allocation within second trial assignment, duration of diabetes, stroke, HbA1c, ethnicity, body mass index, peripheral neuropathy, urine albumin-to-creatinine ratio, and baseline use of insulin	Cross-sectional analyses (unadjusted): association of history of HMA with lower cognitive function at baseline. Lower baseline cognitive function predicted increased risk of first-ever HMA and HAA (but not recurrent HMA or HAA) during follow-up across intervention groups (fully adjusted analyses). Association between 20-month cognitive decline and risk of first-ever HMA in subsequent 22 months (finding restricted to group scoring in lowest tertile of Digit Symbol Coding at baseline; analysis adjusted only for second trial assignment, group allocation within second trial assignment).
Whitmer *et al.* [39] (2009)	Patients with type 2 diabetes; USA	18-year retrospective, observational	17,000	Mean 65 ± 7 years	ICD codes for emergency treatment or hospitalization for hypoglycemia, 1980 to 2002; additional analysis of 1980 to 1985.	ICD codes for any dementia in inpatient and outpatient medical records 2003 to 2007; additional analysis of 2005 to 2007.	Age, sex, education, ethnicity, duration of diabetes, anti-diabetes treatment, duration of insulin use, HbA1c, body mass index, hyperlipidemia, count scores of co-morbidity based on ICD codes for hypertension, cardiovascular disease, stroke, and end-stage renal disease	In 5- and 18-year analyses: hypoglycemia (versus none) 1980 to 2002 or 1980 to 1985 associated with increased risk of subsequent dementia (analyses of dementia 2003 to 2007). Association of ≥2 episodes of hypoglycemia (but not of single episode of hypoglycemia) versus none with increased risk of dementia in analysis of dementia 2005 to 2007. Findings similar for codes of any hypoglycemia and for episodes resulting in hospitalization.
Yaffe *et al.* [37] (2013)	Patients with diabetes participating in Health ABC; USA	12-year prospective, observational	783	Mean 74 ± 3 years	Hospital records identifying hypoglycemia as primary or secondary diagnosis related to hospitalization during follow-up.	Participants cognitively unimpaired at baseline. Identification of dementia cases on the basis of hospital records showing ICD codes for dementia as primary or secondary diagnosis related to hospitalization, or dementia medication on medication inventory during annual visit; MMSE administered at 2-year intervals.	Age, sex, education, ethnicity, diabetes at baseline, insulin use, HbA1c, APOE e4 status, baseline MMSE, MI, stroke, and hypertension	Cross-sectional analysis (unadjusted): association of history of hypoglycemia with lower cognitive function. Prospective analyses: hypoglycemia associated with increased risk of dementia (fully adjusted analysis; survived additional adjustment for slope of MMSE over time). Dementia associated with increased risk of hypoglycemia (analysis adjusted for adjustment variables, minus HbA1c and APOE e4).

*ACCORD-MIND*, Action to Control Cardiovascular Risk in Diabetes-Memory in Diabetes; *ADVANCE*, Action in Diabetes and Vascular Disease: Preterax and Diamicron Modified Release Controlled Evaluation; *APOE*, apolipoprotein; *HAA*, hypoglycemia needing any assistance; *Health ABC*, Health, Aging and Body Composition Study; *HMA*, hypoglycemia requiring medical assistance; *HSH*, health service use for hypoglycemia; *ICD*, International Classification of Diseases; *MCI*, mild cognitive impairment; *MI*, myocardial infarction; *MMSE*, Mini-Mental State Examination; *SH*, severe hypoglycemia; *TIA*, transient ischemic attack

Cross-sectional analyses have reported an association between a history of previous self-reported or medically verified severe hypoglycemia, defined as any episode requiring external help to effect recovery, and cognitive impairment [14, 33, 34] but this could reflect lower cognitive ability in people who go on to experience a higher frequency of severe hypoglycemia. Indeed, in the Action in Diabetes and Vascular Disease: Preterax and Diamicron Modified Release Controlled Evaluation (ADVANCE) trial, which successfully manipulated the level of glycemic control (intensive versus standard) in patients with type 2 diabetes, each one-unit-lower score on a cognitive screening instrument at baseline was associated with a 10 % greater risk of severe hypoglycemia during follow-up [35]. A lower baseline cognitive function and (for participants who had low processing speed at baseline) a relatively steeper cognitive decline between baseline and the 20-month assessment were also predictive of an increase in the subsequent first-ever hospital admission to treat severe hypoglycemia in ACCORD-MIND, and the group with low processing speed and declining cognitive function had a higher cumulative incidence of severe hypoglycemia over the 4 years of the study [36]. Finally, in two further prospective investigations, a baseline diagnosis of dementia or a diagnosis during the follow-up period in previously unimpaired participants was associated with a two- to three-fold higher rate of hospital admission for emergency medical treatment of hypoglycemia during follow-up [34, 37].

**Fig. 2:**
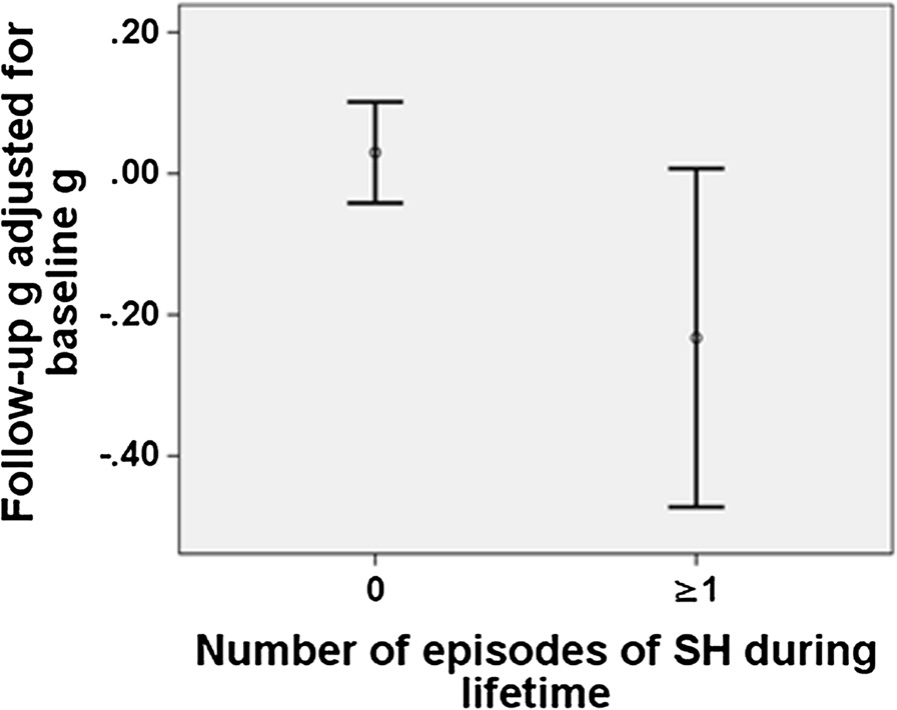
Relationship of severe hypoglycemia with cognitive decline in the Edinburgh Type 2 Diabetes Study. Relationship of a self-reported history of one or more episodes of severe hypoglycemia (SH) (defined as requiring external help) prior to the baseline clinic of the Edinburgh Type 2 Diabetes Study with the subsequent decline on a composite measure (‘g’) of cognitive ability during 4-year follow-up. Error bars show 95 % confidence interval (n = 831). Based on [38]

Whether exposure to hypoglycemia precedes cognitive decline and may even be a causal risk factor for this condition is less clear. In the Edinburgh Type 2 Diabetes Study (ET2DS) of more than 1,000 adults between 60 and 75 years of age, a history of severe hypoglycemia was associated with lower cognitive function when the estimated pre-morbid cognitive function before exposure to hypoglycemia was compared with post-hypoglycemia cognitive function, with evidence of an acceleration of late-life cognitive decline that was independent of the potential influence of further episodes of hypoglycemia [38] (Fig. 2). However, these observations conflict with the findings of the Fremantle Diabetes Study [21] and with the evidence from RCTs. In ACCORD-MIND and ADVANCE, cognitive function declined at similar rates by 40 months and 5 years in patients in the intensive treatment groups (in whom the incidences of hypoglycemia were significantly higher) compared with those in the standard treatment arms [28, 35]. However, in both of these trials, the management of diabetes was manipulated to attain pre-determined glycemic targets. It is plausible that any detrimental effect of hypoglycemia was counterbalanced by an improvement in cognitive function occurring through specific beneficial effects of the assigned intervention.

Two retrospective investigations [39, 40] have suggested that a dose-response relationship may exist between the frequency of exposure to severe hypoglycemia and the subsequent risk of dementia. However, these studies relied on hospital records, a suboptimal method of identifying hypoglycemia, and the suggestion that exposure to a single episode of hypoglycemia would induce dementia is biologically implausible. In the observational analysis of the Fremantle Diabetes Study, a history of severe hypoglycemia failed to predict the 5-year risk of dementia [34]. In the prospective Health Aging and Body Composition Study, participants with incident hypoglycemia had a two-fold risk of subsequent dementia over a period of 12 years, but in combination with the analysis showing an increased risk of subsequent hypoglycemia in patients who were diagnosed with dementia during follow-up, the data were overall interpreted as showing bidirectional causality [37]. It is essential that the role of hypoglycemia in either causing or accelerating cognitive decline be clarified in view of the current policy to use intensive therapy to achieve near-normoglycemia to minimize the development of diabetic complications.

### Hyperinsulinemia

Hyperinsulinemia from endogenous hypersecretion of insulin is common in the early stages of type 2 diabetes as a ‘pathophysiological’ response to insulin resistance; it also occurs as a consequence of exogenous insulin therapy. Hyperinsulinemia has been associated with cognitive impairment, but a systematic review of observational studies that included people with and those without diabetes concluded that the evidence for an association of elevated plasma insulin concentrations with impairment of cognition was weak, because it is possible that any association of plasma insulin with cognition in such samples had been influenced by the inclusion of people with diabetes [24]. Very few studies have been performed in non-diabetic populations or exclusively in people with type 2 diabetes (Table 3). In one observational study, a higher mean insulin during a 6-year period was associated with a steeper rate of concurrent cognitive decline based on a test of executive function [16], and in a small intervention study, which was unsuccessful in inducing a difference in insulin sensitivity in two treatment groups through physical exercise, improvement in memory performance correlated with improvements in insulin resistance [15]. By contrast, in a larger 24-week trial in middle-aged to older patients with type 2 diabetes (mean age of 60 years), an improvement in plasma insulin levels and insulin sensitivity had no effect on concurrent change in cognitive function [29]. Similarly, in the ACCORD-MIND study, treatment with insulin on study entry or during the trial was relatively unrelated to 40-month cognitive change, but plasma insulin levels as such were not considered [41]. This is despite the fact that compliance by participants is difficult to ascertain. Inter-relationships between plasma insulin concentration, insulin resistance, and the quality of glycemic control further complicate attempts to evaluate associations of any of these risk factors with cognitive impairment.

### Inflammation

Chronic low-grade inflammation is a characteristic feature of both diabetes and AD and appears to interact with diabetes in its association with cognitive impairment. This suggests a common biological mechanism [42]. Circulating markers of inflammation include C-reactive protein (CRP), interleukin-6 (IL-6), fibrinogen, and tumor necrosis factor-alpha (TNF-α), some of which have been associated with cognitive dysfunction in people with diabetes (Additional file 1: Table S1). Elevated levels of CRP have been associated with lower cognitive function in small studies of hospitalized patients (for example, [9]). In the ET2DS, higher levels of fibrinogen, TNF-α, and IL-6 but not CRP were associated with lower measures of cognitive function [43, 44]; higher baseline levels of fibrinogen and IL-6 additionally predicted a steeper 4-year cognitive decline [45, 46]. CRP levels were also unrelated to cognitive decline in a further prospective study with a 6-year follow-up [16]. In support of associations (particularly, causal) between inflammation and cognition, genetic variants that influence circulating levels of inflammatory markers have been associated with cognitive impairment, but this finding has not been consistent [43, 47].

### Microvascular disease

Because of the homology between retinal and cerebrovascular cells, the state of small vessels in the retina closely mirrors that of the cerebral microvasculature, suggesting that diabetic retinopathy can be used as a marker for the presence of microangiopathy within the brain. A systematic review of cross-sectional and prospective observational studies concluded that people from the general population and people with diabetes who exhibit retinal microvascular abnormalities appear to be at increased risk of cognitive impairment, including dementia, compared with people who have no retinal microvascular abnormalities [48], although subsequent studies have given conflicting results [25, 35, 49] (Additional file 1: Table S2). However, in support of the findings of the systematic review, baseline presence of retinopathy was recently identified as a predictor of steeper rates of cognitive decline during 40-month (but not 20-month intermittent) follow-up in ACCORD-MIND [50]. Overall, diabetic retinopathy may be a putative surrogate marker for cognitive impairment in people with diabetes, in which cerebral microvascular disease may have an important pathogenetic role.

## Macrovascular disease

The prevalence of both symptomatic and asymptomatic macrovascular disease is increased in people with type 2 diabetes. Given the likely links between vascular and cognitive pathologies, markers of such vascular ‘end-organ damage’ have the potential to identify a group of subjects who are at particularly high risk of developing cognitive impairment. Assessing the association between different macrovascular diseases and cognitive impairment may also help us understand underlying pathophysiological mechanisms. To this end, it is interesting to consider the extent to which studies have uncovered associations of cognitive impairment with specific types of macrovascular disease, such as coronary heart disease (CHD), cerebrovascular disease, and peripheral arterial disease, and with vascular biomarkers which indicate underlying subclinical macrovascular disease in the related vascular trees.

### Coronary heart disease and N-terminal pro-brain natriuretic peptide

An association of CHD with a lower level of cognitive function was observed in the ET2DS [51] (Additional file 1: Table S3). However, in the ACCORD-MIND study, the evidence for an association of CHD with cognitive dysfunction was limited [13], and in all other cross-sectional investigations [14, 35] and in all prospective analyses, including the ET2DS [21, 51], the results have been negative. The only significant prospective association was in the direction of cognitive ability predicting worsening of CHD. In ADVANCE, after multivariate adjustment, a baseline presence of ‘mild cognitive dysfunction’ and ‘severe cognitive dysfunction’ increased the 5-year risk of a major coronary event by 31 % and 70 %, respectively [35].

The inactive metabolite N-terminal pro-brain natriuretic peptide (NT-proBNP) is a biomarker of the cardiac stress associated with ventricular dysfunction and congestive heart failure. In the ET2DS, an association of small effect size was found between a higher baseline NT-proBNP and a lower cognitive ability and with a steeper cognitive decline later in life [51] (Additional file 1: Table S4). In the general population and in people with cardiovascular disease, associations of small to large unadjusted effect size have been reported relatively consistently between elevated levels of natriuretic peptide and lower cognitive function (for example, [52]) and with the presence of dementia or milder forms of impairment [53, 54]. Null findings are rare [55] and in some studies may have resulted from the nature of the cognitive screening instruments that were applied. Some results have suggested an independence of these associations from symptomatic macrovascular disease, including stroke [52], which was also observed in the ET2DS [51]. Prospective investigations of the general population were, until recently, restricted to a single cohort (of people over 75 years of age) in which the findings were inconclusive [53, 54]. However, a recent large Finnish study (n = 7,000 participants) that examined the relationship of natriuretic peptides with cognitive function has not provided definitive evidence [56]. In that study, each standard deviation above baseline NT-proBNP predicted a 48 % increased risk of dementia during 14-year follow-up after multivariate adjustment in men, but no such association was found in women.

### Cerebrovascular disease and carotid intima-media thickness

An association between a lower level of cognitive function and cerebral infarction has been a consistent finding in populations with diabetes [13, 51] (Additional file 1: Table S3). For example, in the Fremantle Diabetes Study, cognitive impairment diagnosed on the basis of a screening instrument and follow-up clinical interview was associated with a history of cerebrovascular disease [14]. The impact of stroke on cognitive function was demonstrated in the diabetic subpopulation of a Dutch study and in the ET2DS, in which an association between stroke and diminished cognitive function persisted after adjustment for estimated pre-morbid ability [20, 25, 51].

In the ET2DS and in the Fremantle Diabetes Study, a history of stroke was associated with a steeper decline in cognitive ability [14, 51], but this observation differed from those of several other prospective analyses (for example, [21]), in which no such association was found. In the ADVANCE study, evidence of a prospective association in the direction of lower cognitive function predisposing patients to an increased risk of infarction was observed. After multivariate adjustment, individuals with ‘mildly reduced’ cognitive function at baseline had a 5-year risk of sustaining a major stroke which was 34 % greater than that of individuals who had a higher level of cognitive ability; people with ‘severe cognitive dysfunction’ had a 71 % greater risk [35]. The relationship between cerebral infarction and cognition in diabetes may therefore be bidirectional.

In people with type 2 diabetes, greater carotid intima-media thickness (cIMT) has been associated with a lower level of cognitive function [9, 11], but its association with an estimated steeper decline of lifetime cognitive function has been inconsistent [25, 51] (Additional file 1: Table S3). To date, the ET2DS appears to be the only prospective study to examine cIMT and cognition in people with type 2 diabetes. This identified an association of cIMT with a steeper decline in late-life cognitive function, which was independent of a preceding history of stroke [51]. In the population in general, an association between a higher cIMT and an increased risk of cognitive impairment has been established [57], and so a similar association is likely to exist in people with type 2 diabetes.

### Peripheral arterial disease and ankle-brachial pressure index

In people with type 2 diabetes, a low ankle-brachial pressure index (ABI) - a measure of peripheral arterial disease (PAD) of the lower limbs and of more generalized atherosclerosis - and PAD diagnosis have been associated with lower cognitive function [9, 51] and with dementia [14] (Additional file 1: Table S3). In the Fremantle Diabetes Study, 38 % of cognitively ‘normal’ individuals, 45 % of people with reduced cognitive function, and 75 % of people with frank dementia had evidence of coexisting PAD [14]. In one study, cross-sectional findings for ‘any vascular event’ (which was partly defined by PAD) remained significant after adjustment for an estimate of peak pre-morbid ability [20, 25], but after such an adjustment was made in the analysis of ABI and symptomatic PAD in the ET2DS, it did not quite achieve statistical significance [51]. However, in the latter, each standard deviation of a lower baseline ABI was also associated with a 0.12-standard deviation increment in subsequent 4-year decline on a composite measure of cognitive function [51]. In the Fremantle Diabetes Study, PAD measured 8 years earlier also predicted an increased risk of cognitive impairment [14] though it was not associated with the risk of cognitive decline in the subsequent 2-year follow-up period [21].

Overall, the evidence for an association between macrovascular disease and cognitive impairment in diabetes is inconsistent and varies according to the area of the vasculature considered. As might be expected, evidence for a relationship with cerebrovascular disease, especially stroke, is stronger than that for vascular sites which are more distant from the brain, including the heart. Evidence for an association with the most distal presentations of macrovascular disease, such as PAD of the lower limbs, is particularly limited, is likely to reflect widespread atherosclerosis as a marker for cognitive impairment in people with diabetes, and would suggest that any true associations have a small effect size.

## Depression and pre-morbid cognitive ability

### Depression

Cross-sectional studies of cognitive function in people with diabetes, with or without depressive symptoms or clinical depression, have been inconclusive (Additional file 1: Table S5). One investigation of older people with type 2 diabetes reported a statistically non-significant trend for negative correlations between scores on a cognitive screening instrument and scores on a self-administered screening instrument for depression [58]. In a cross-sectional analysis of ACCORD-MIND, patients with depression (based on scores on screening instrument or on self-report) also scored lower on a cognitive screening instrument (though not on more detailed neuropsychological tests) compared with patients who were free of depression [13]. Additive detrimental effects have been suggested by another study of people with type 2 diabetes and healthy controls who were 30 to 80 years of age (the mean age was 60 years across groups), to whom more detailed neuropsychological testing was applied along with clinical interviews to diagnose depression. The patients with co-morbid diabetes and depression performed less well on tests of attention and processing speed compared with participants with diabetes but without depression. Relative to the latter, there was also a trend just short of statistical significance for lower cognitive function overall in the group with co-morbid diabetes and depression [59]. In a prospective analysis of a large cohort of Americans, co-morbidities of diabetes and depression were also linked to a 100 % increased risk of dementia over a period of 3 to 5 years when compared with people with diabetes but without depression [60]. Finally, ACCORD-MIND revealed associations of higher scores on a screening instrument for depression and a steeper 40-month cognitive decline [61]. In the general population, the association of depression with cognitive impairment appears to be well established [60], and so it seems likely that depression has a contributing role in promoting diabetes-associated cognitive impairment.

### Pre-morbid cognitive ability

**Fig. 3:**
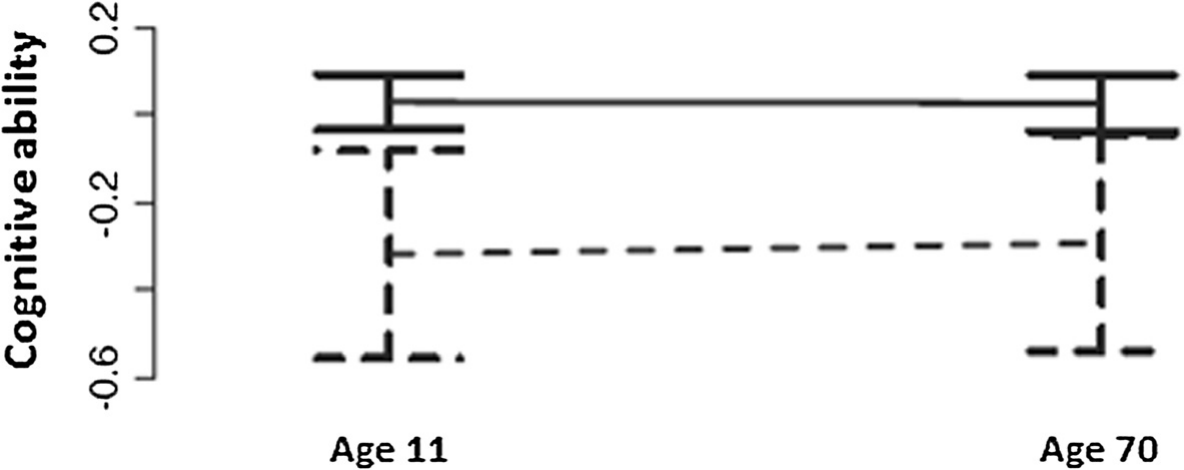
Mean difference (95 % confidence interval) in cognitive test scores in people with and without diabetes at ages 11 and 70 in the Lothian Birth Cohort (1936). Solid lines reflect people without diabetes at age 70; dashed lines reflect people with diabetes at age 70. Reproduced with permission from Elsevier [62]

Diabetes-associated cognitive impairment may partly reflect reverse causality. Consistent with the assumption that individuals who have lower cognitive ability may be predisposed to have lower late-life cognitive function and to be at increased risk of developing diabetes as they get older, an analysis of the Lothian Birth Cohort (a group of people who were born in 1936) found that cross-sectional associations of diabetes with lower late-life cognitive ability disappeared following adjustment for cognitive ability that had been measured at age 11 [62] (Fig. 3).

However, where diabetes is associated with a steeper late-life cognitive decline in prospective analyses, the role of pre-morbid ability is as yet unclear, particularly as its role in promoting late-life cognitive decline *per se* is uncertain. Some prospective investigations have indicated that individuals with lower cognitive ability decline more rapidly as they get older [63], but this has not been confirmed [64].

## Neuropathological effects

The neuropathological features of VaD (multiple infarcts) and AD (cerebral plaques of beta amyloid and hyperphosphorylated tau contributing to neurofibrillary tangles) [65] are well established. Increasingly, it is being recognized that there may be considerable overlap in the etiology of these two conditions [66, 67], and individuals with cognitive decline often exhibit both pathologies. Many of the risk factors reviewed in this article have the potential to contribute to such neuropathology. Clearly, it is not difficult to conceive how the macrovascular risk factors in diabetes would contribute to cerebrovascular damage [12], while chronic hyperglycemia may lead to the accumulation of advanced glycation end-products in the brain [68] and the development of small vessel ischemic change. The neurotoxic effects of hypoglycemia are also well understood [8], and there are strong links between insulin and beta amyloid: insulin appears to initiate the production of beta amyloid as well as promote its accumulation through competition for degradation by insulin-degrading enzyme [67]; it may further contribute to amyloid formation through co-secretion of the amyloid-forming peptide amylin with insulin from pancreatic beta cells [65, 69]. Consistent with amylin being a neuropathological mediator of associations between diabetes and cognitive impairment, a recent post-mortem study demonstrated the presence of the peptide in the brains of people with diabetes and in those with AD, but not in healthy controls [69]. Additional associations of amylin with vascular damage [69] are consistent with the premise that AD and VaD may not be as clearly distinct as has been thought previously.

As becomes clear, the neuropathological bases of the increased risk of cognitive impairment that people with diabetes are exposed to are far from singular and straightforward. Rather, highly complex, cell-level processes appear to be at play. It is this complexity which explains the difficulty in the development of effective strategies for prevention of cognitive impairment in people with diabetes and in the development of treatment approaches in those patients who have already become cognitively impaired.

## Conclusions

Most studies that have addressed the risk factors associated with cognitive impairment have examined cohorts from the general population. However, in view of the greater risk of cognitive impairment affecting people with type 2 diabetes and the potential differences in underlying mechanisms between people with type 2 diabetes and the general population, more information that is specific to diabetic populations is required, particularly in older adults. The evidence that risk factors that occur more frequently in people with type 2 diabetes are associated with cognitive impairment is limited, mainly because few of these risk factors have been investigated in any depth. Many have also been assessed in isolation. The evidence that is currently available points to a role for poor glycemic control, hypoglycemia, micro- and macrovascular disease, inflammation, and depression as potential risk factors for cognitive impairment in people with diabetes. However, the causality in these relationships is less clear. The roles of dyslipidemia, hyperinsulinemia, hypertension, and pre-morbid ability as putative risk factors are as yet undetermined and require further investigation. Overall, we would recommend that clinicians temper the current emphasis on intensive therapy and strict glycemic control in an attempt to protect the cognitive function of their patients (particularly in view of the potentially detrimental effects that hypoglycemia may have on cognition). We would encourage them to take a holistic approach to patient management by addressing the full range of modifiable risk factors while being aware of the potential influences of risk factors for cognitive impairment that are not modifiable.

A previous review of research in this field [70] has indicated that evidence has advanced mainly in a quantitative manner in recent decades. For modifiable risk factors, further high-quality and large-scale trials are needed to determine causality in the interaction between each major risk factor and their association with cognitive decline. For glycemic control, future trials should continue to attempt to separate out the potential duality of beneficial (reduced blood glucose levels) and detrimental (hypoglycemia) effects. Rather than using statistical adjustment methods, such as controlling for hypoglycemia in analyses of anti-diabetes agents and cognitive decline, effects of anti-diabetes agents that do not induce hypoglycemia could be investigated for that purpose.

Novel directions could also be taken to investigate risk factors for which the evidence has been largely restricted to observational studies despite being modifiable. For instance, trials could determine effects of anti-inflammatory medication such as non-steroidal anti-inflammatory drugs, which are already relatively widely used and are low in cost, in order to provide definitive evidence on potential associations of these risk factors with cognitive impairment in people with diabetes as have become apparent from some observational investigations.

Undoubtedly, large trials are difficult and costly to conduct, not least because they are resource-intensive, and for non-modifiable risk factors are not always possible. As a consequence, cohort studies are likely to continue dominating this field of research. Harmonization of risk factor assessments and methodologies between cohorts should be sought with the aim of enabling integration of a range of cohorts into single large-scale analyses. Instead of focusing on individual risk factors with resultant data ‘slicing’, investigators should ascertain the inter-relationships among a range of risk factors and should explore their temporal developments. Specifically, future cohort studies, including birth cohorts, could use multi-wave designs to allow statistical procedures such as latent growth curve modeling to determine the probable inter-relationships among putative risk factors and establish their true associations (if any) with cognitive decline. In view of recent evidence of an association between cognitive impairment and brain atrophy in mid-life diabetes [71], the age at which individuals are recruited for cohort studies may have to be reconsidered to enable a life-course approach to this issue. It is to be hoped that ongoing and future research will identify causal risk factors that can be used to develop preventative interventions and help to identify which patients are at greatest risk of developing cognitive impairment.

## Additional file

Additional file 1: Tables S1-S5. A summary of the evidence reviewed in this article on associations of inflammation, microvascular disease, macrovascular disease, natriuretic peptides and depression with cognitive function.

## Abbreviations

ABI: ankle-brachial pressure index
ACCORD-MIND: Action to Control Cardiovascular Risk in Diabetes-Memory in Diabetes
AD: Alzheimer’s disease
ADVANCE: Action in Diabetes and Vascular Disease: Preterax and Diamicron Modified Release Controlled Evaluation
CHD: coronary heart disease
cIMT: carotid intima-media thickness
CRP: C-reactive protein
ET2DS: Edinburgh Type 2 Diabetes Study
IL-6: interleukin-6
NT-proBNP: N-terminal pro-brain natriuretic peptide
PAD: peripheral arterial disease
RCT: randomized controlled trial
TNF-α: tumor necrosis factor alpha
VaD: vascular dementia
