# Supplementary material for: The impact of diabetes on cognitive decline: potential vascular, metabolic, and psychosocial risk factors
Source: Alzheimers Res Ther. 2015 Jun 10;7(1):46. doi: 10.1186/s13195-015-0130-5 (PMC4460635; doi:10.1186/s13195-015-0130-5)
Supplement: Additional file 1: Tables S1-S5. — A summary of the evidence reviewed in this article on associations of inflammation, microvascular disease, macrovascular disease, natriuretic peptides and depression with cognitive function. [file 13195_2015_130_MOESM1_ESM.docx]

Supplemental Data

{2 Column Table}

**Table S1. Studies of inflammation and cognitive function in type 2 diabetes**

| Study | Sample | Design | Number | Baseline mean age | Measurement of inflammation | Cognitive measures | Adjustment variables | Association with cognitive function |
| --- | --- | --- | --- | --- | --- | --- | --- | --- |
| Chen *et al*. [9] (2011) | Patients with type 2 diabetes;  China | Cross-sectional, observational | 101 | Mean 63 ± 8 years | CRP | MCI identified on the basis of cognitive screening instrument | None | Higher CRP in group with MCI compared with group free of MCI. |
| Keller *et al*. [45] (2012) | Patients with type 2 diabetes participating in the Edinburgh Type 2 Diabetes Study;  Scotland | 4-year prospective, observational | 1,066 | Mean 68 ± 4 years | Plasma fibrinogen at baseline | Composite score from seven cognitive tests | Age and sex | Higher baseline fibrinogen associated with steeper cognitive decline |
| Keller *et al*. [46] (2012) | Patients with type 2 diabetes participating in the Edinburgh Type 2 Diabetes Study;  Scotland | 4-year prospective, observational | 1,066 | Mean 68 ± 4 years | Plasma IL-6 at baseline | Composite score from seven cognitive tests | Age and sex | Higher baseline IL-6 associated with steeper cognitive decline. |
| Marioni *et al*. [43] (2011) | Patients with type 2 diabetes participating in the Edinburgh Type 2 Diabetes Study;  Scotland | Cross-sectional, observational | 1,066 | Mean 68 ± 4 years | Plasma fibrinogen | Composite score from seven cognitive tests | Age and sex | Higher fibrinogen associated with lower cognitive function. |
| Marioni *et al*. [44] (2010) | Patients with type 2 diabetes participating in the Edinburgh Type 2 Diabetes Study;  Scotland | Cross-sectional, observational | 1,066 | Mean 68 ± 4 years | Plasma IL-6, TNF-α, and CRP | Composite score from seven cognitive tests and estimate of pre-morbid ability | Age, sex, education, cardiovascular disease, duration of diabetes, HbA1c, and estimated pre-morbid ability | Higher IL-6 associated with lower cognitive function and steeper estimated lifetime decline (fully adjusted analyses). Association of higher TNF-α with lower cognitive function and estimated lifetime decline only in analyses adjusted for age, sex, and estimated pre-morbid ability. No consistent finding for CRP. |
| Umegaki *et al*. [16] (2014) | Patients with type 2 diabetes;  Japan | 6-year prospective, observational | 79 | Mean 74 ± 5 years | Mean of CRP measured at baseline and annual follow-ups | Composite score from MMSE, Digit Symbol Coding, Stroop, and word recall. Analyses of ‘decliners’ versus ‘non-decliners’ on bases of composite score and individual cognitive tests. | None | No association. |

CRP, C-reactive protein; IL-6, interleukin-6; MCI, mild cognitive impairment; MMSE, Mini-Mental State Examination; TNF-α, tumor necrosis factor-alpha.

{2 Column Table}

**Table S2. Studies of microvascular disease and cognitive function in type 2 diabetes**

| Study | Sample | Design | Number | Baseline mean age | Measurement of microvascular disease | Cognitive measures | Adjustment variables | Association with cognitive function |
| --- | --- | --- | --- | --- | --- | --- | --- | --- |
| de Bresser *et al*. [49] (2010) | Patients with type 2 diabetes participating in the Utrecht Diabetic Encephalopathy Study;  The Netherlands | 4-year prospective, observational | 122 | Mean 66 ± 6 years | Ophthalmologist-identified presence versus absence of retinopathy according to ‘standard clinical practice’ at baseline | Composite score from 11 cognitive tests, estimate of pre-morbid ability, total brain volume, lateral ventricular volume, white matter hyperintensity volumes, and cerebral infarcts | Age, sex, and pre-morbid ability for analyses of cognitive function. Age and sex for analyses of imaging data. | No association. |
| de Galan  *et al*. [35] (2009) | Patients with type 2 diabetes participating in ADVANCE arm on glycemic control, receiving standard target versus target HbA1c ≤6.5%;  Australia | 5-year trial on effects of intensified blood pressure control and intensified glycemic control | 11,140 | Mean 66 ± 6 years | ‘Major diabetic eye disease’ | At baseline and 2-year intervals: MMSE followed by clinical interview for patients with MMSE <24 or suspected dementia. ‘Normal’ cognitive function defined as MMSE ≥28; ‘mild dysfunction’ as MMSE = 24-27; ‘severe dysfunction’ as MMSE <24. Additional use of MMSE as continuous measure. | None | Cross-sectional analysis: increased prevalence of major diabetic eye disease in groups with cognitive dysfunction. |
| Hugenschmidt *et al*. [50] (2014) | Patients with type 2 diabetes participating in ACCORD-Eye and ACCORD-MIND substudies of ACCORD; North America | 40-month prospective, observational | 1,862 | Mean 62 ± 6 years | Presence of no retinopathy, of mild non-proliferative retinopathy, or of moderate/severe retinopathy at baseline; based on ETDRS chart | Digit Symbol Coding (primary outcome), MMSE, Rey Auditory Verbal Learning, and Stroop (secondary outcomes) at baseline and 20 and 40 months. Total brain volume, white matter volume, gray matter volume, and abnormal white matter volume at baseline and 40 months. | Age, sex, ethnicity, education, smoking, geographic region, duration of diabetes, HbA1c, cholesterol, triglycerides, blood pressure, anti-hypertensive medication use, depression, alcohol, and neuropathy. Additional adjustment for visual acuity for analyses of cognitive function and for total intracranial volume for analyses of brain volumes. | Cross-sectional association of retinopathy with lower gray matter volume but not with cognitive function. Prospective association of retinopathy with steeper 40-month (but not 20-month) decline on MMSE and Digit Symbol Coding. Statistically non-significant trend for association of baseline retinopathy with greater increase in white matter abnormalities during 40-month follow-up. |
| Manschot *et al*. [25] (2007) | Patients with type 2 diabetes participating in the Utrecht Diabetic Encephalopathy Study;  The Netherlands | Cross-sectional, observational | 122 | Mean 66 ± 6 years | Presence versus absence of retinopathy based on scores on diabetic retinopathy severity scale (Wisconsin Epidemiologic Study of Diabetic Retinopathy) at baseline | Composite score from 11 cognitive tests, estimate of pre-morbid ability, cortical atrophy, and white matter lesions | Age, sex, and estimated pre-morbid ability | No association of retinopathy with estimated lifetime decline in cognitive function. Association of retinopathy with presence of cortical atrophy (analysis additionally controlling for lipid-lowering drugs and cerebral infarcts). |

ACCORD, Action to Control Cardiovascular Risk in Diabetes; ACCORD-MIND, Action to Control Cardiovascular Risk in Diabetes-Memory in Diabetes; ADVANCE, Action in Diabetes and Vascular Disease: Preterax and Diamicron Modified Release Controlled Evaluation; ETDRS, Early Treatment of Diabetic Retinopathy Study; MMSE, Mini-Mental State Examination.

{2 Column Table}

**Table S3. Studies of markers of macrovascular disease and cognitive function in type 2 diabetes**

| Study | Sample | Design | Number | Baseline mean age | Macrovascular disease | Cognitive measures | Adjustment variables | Association with cognitive function |
| --- | --- | --- | --- | --- | --- | --- | --- | --- |
| Bruce *et al*. [14] (2008) | Patients with type 2 diabetes participating in the Fremantle Diabetes Study; Australia | 8-year retrospective, observational | 302 | Mean 76 ± 5 years | ‘Cerebrovascular disease’ (stroke and TIA), ‘CHD’ (MI, angina, coronary artery bypass, and evidence of MI on ECG), and peripheral arterial disease at baseline and 8 years earlier | Dementia and MCI identified from screening instruments/clinical interview | None | Cross-sectional analyses: increased prevalence of cerebrovascular disease across cognitive groups, with highest prevalence in dementia group, followed by MCI group and unimpaired group. Increased prevalence of PAD in group with any cognitive impairment (in model controlling for sex, duration of diabetes, and stroke) and in group with dementia (in model controlling for age, sex, and duration of diabetes).  Prospective analyses: cerebrovascular disease (unadjusted analysis) and PAD (model controlling for age, sex, duration of diabetes, and waist-hip ratio) 8 years earlier both associated with poorer cognitive outcome. No findings for CHD. |
| Bruce *et al*. [21] (2008) | Patients with type 2 diabetes participating in the Fremantle Diabetes Study; Australia | 8-year retrospective, 2-year prospective, observational | 205 | Mean 75 ± 4 years | Cerebrovascular disease and peripheral arterial disease assessed 8 years prior to baseline cognitive assessment | Dementia and MCI identified from screening instruments/clinical interview at baseline and at 2-year follow-up. ‘Cognitive decline’ defined as downward conversion between ‘normal’, MCI, and dementia. | None | No association. |
| Chen *et al*. [9] (2011) | Patients with type 2 diabetes;  China | Cross-sectional, observational | 101 | Mean 63 ± 8 years | ABI and cIMT | MCI identified on the basis of screening instrument | None | Lower ABI and higher cIMT in group with MCI compared with group free of MCI. |
| Chen *et al*. [11] (2012) | Patients with type 2 diabetes;  China | Cross-sectional, observational | 157 | Mean 55 ± 7 years | cIMT | MCI identified on the basis of cognitive screening instrument | None | Higher cIMT in group with MCI compared with group free of MCI. Inverse correlation between cognitive scores and cIMT. |
| Cukierman-Yaffe  *et al*. [13] (2009) | Patients with type 2 diabetes participating in ACCORD-MIND;  North America | Cross-sectional analysis of trial on blood pressure, lipids, and glycemic control | 2,977 | Mean 63 ± 6 years | Stroke, ‘CVD’ (stroke, MI, angina with ischemic changes, and coronary procedure) | Digit Symbol Coding (primary outcome), MMSE, Rey Auditory Verbal Learning, and Stroop (secondary outcomes) | Age | Association of stroke with lower cognitive function. Association of CVD (without stroke) with lower verbal memory but higher MMSE scores. |
| de Galan *et al*. [35] (2009) | Patients with type 2 diabetes participating in ADVANCE arm on glycemic control; Australia | 5-year prospective trial on effects of intensified blood pressure control and intensified glycemic control | 11,140 | Mean 66 ± 6 years | Stroke, MI, major coronary event (non-fatal MI, death from coronary event), and major CVD event (MI, stroke, and cardiovascular death) during follow-up | At baseline and 2-year intervals: MMSE followed by clinical interview for patients with MMSE <24 or suspected dementia. ‘Normal’ cognitive function defined as MMSE ≥28; ‘mild dysfunction’ as MMSE = 24-27; ‘severe dysfunction’ as MMSE <24. Additional use of MMSE as continuous measure. | Age, sex, education, and treatment allocation | Cross-sectional, unadjusted analyses: association of stroke and major CVD event with lower cognitive function. No finding for MI. Prospective analyses (adjusted):  baseline mild and severe impairment (versus unimpaired) associated with increased risk of major CVD event, stroke, and major coronary event during follow-up. Lower MMSE at baseline associated with increased risk of major CVD event. |
| Feinkohl *et al*. [51] (2013) | Patients with type 2 diabetes participating in the Edinburgh Type 2 Diabetes Study;  Scotland | 4-year prospective, observational | 1,066 | Mean 68 ± 4 years | MI, stroke, angina, ABI, and cIMT at baseline | MMSE, composite score from seven cognitive tests, and estimate of pre-morbid ability | Age, sex, cholesterol, blood pressure, smoking, and estimated pre-morbid ability | Cross-sectional analyses: stroke, angina, and MI associated with lower cognitive function. Prospective analyses: stroke, higher cIMT, and lower ABI associated with increased late-life cognitive decline and increased estimated lifetime decline. |
| Manschot *et al*. [20]  (2006) | Patients with type 2 diabetes participating in the Utrecht Diabetic Encephalopathy Study;  The Netherlands | Cross-sectional, observational | 122 | Mean 66 ± 6 years | ‘Any macrovascular event’ defined as MI, stroke, or surgery/endovascular treatment for coronary, carotid or peripheral arterial disease.  Cerebral infarcts on MRI. | Composite scores on five cognitive domains from 11 cognitive tests, estimate of pre-morbid ability, cortical atrophy, and white matter lesions | Age, sex, and estimated pre-morbid ability | Association of infarcts with steeper estimated lifetime decline in processing speed. Association of any macrovascular event with steeper estimated lifetime decline in processing speed and memory. |
| Manschot *et al*. [25] (2007) | Patients with type 2 diabetes participating in the Utrecht Diabetic Encephalopathy Study;  The Netherlands | Cross-sectional, observational | 122 | Mean 66 ± 6 years | ‘Any macrovascular event’ (MI, stroke, and surgery/endovascular treatment for coronary, carotid, or peripheral arterial disease) and cerebral infarcts on MRI. Measurement of cIMT. | Composite score from 11 cognitive tests, estimate of pre-morbid ability, cortical atrophy, and white matter lesions | Age, sex, and estimated pre-morbid ability | ‘Any macrovascular event’ and infarcts both associated with steeper estimated lifetime cognitive decline. Finding on ‘any vascular event’ attenuated following exclusion of patients with stroke. For ‘any vascular event’, but not infarcts, association persisted in final model adjusting for age, estimated pre-morbid ability, hypertension, smoking, and lipid-lowering drugs. No finding for cIMT. |

ABI, ankle brachial index; ACCORD-MIND, Action to Control Cardiovascular Risk in Diabetes-Memory in Diabetes; ADVANCE, Action in Diabetes and Vascular Disease: Preterax and Diamicron Modified Release Controlled Evaluation; CHD, coronary heart disease; cIMT, carotid intima-media thickness; CVD, cardiovascular disease; ECG, electrocardiogram; MCI, mild cognitive impairment; MI, myocardial infarction; MMSE, Mini-Mental State Examination; MRI, magnetic resonance imaging; PAD, peripheral arterial disease; TIA, transient ischemic attack.

{2 Column Table}

**Table S4. Studies of natriuretic peptides and cognitive function in the general population and in type 2 diabetes**

| Study | Sample | Design | Number | Baseline mean age | Natriuretic peptide | Cognitive measures | Adjustment variables | Association with cognitive function |
| --- | --- | --- | --- | --- | --- | --- | --- | --- |
| Daniels *et al*. [52] (2011) | Rancho Bernardo Study; USA | Cross-sectional, observational | 950 | Mean 77 ± 8 years | NT-proBNP, categorized as ‘high’ (≥450 pg/mL) versus ‘low’ (<450 pg/mL) and additionally as quartiles of the distribution | MMSE, Trail-Making Test, and Category Fluency.  ‘Low’ performance on each defined as MMSE ≤24, Trail-Making ≥300 seconds, and Category Fluency ≤12 words. Cognitive tests additionally used as quartiles of respective distribution. | Age, sex, education, hypertension, body mass index, exercise, alcohol, smoking, cholesterol, and creatinine | ‘High’ NT-proBNP group likely to score low on MMSE and Trail-Making compared with ‘low’ group. Findings similar when excluding participants with stroke and cardiovascular disease. Findings for Category Fluency restricted to largely unadjusted analyses. Additionally increased prevalence of scoring in lower quartiles of cognitive scores with increasing quartiles of NT-proBNP (unadjusted analyses). |
| Feinkohl *et al*. [51] (2013) | Patients with type 2 diabetes participating in the Edinburgh Type 2 Diabetes Study;  Scotland | 4-year prospective, observational | 1,066 | Mean 68 ± 4 years | NT-proBNP at baseline | MMSE, composite score from seven cognitive tests, and estimate of pre-morbid ability | Age, sex, cholesterol, blood pressure, and smoking | Association of higher baseline NT-proBNP with steeper late-life cognitive decline and steeper estimated lifetime decline. Finding on 4-year decline lost statistical significance when controlled for estimated pre-morbid ability or for stroke, cIMT, and ABI. |
| Kerola *et al*.  [53] (2010) | Kuopio 75+ cohort;  Finland | 5-year prospective, observational | 464 | Mean 80 ± 4 years | BNP at baseline | Diagnosis of AD, VaD, and MMSE | Age, education, and hypertension | Association of higher BNP with lower MMSE and with steeper decline on MMSE (unadjusted analyses).  Association of higher BNP and increased risk of AD and VaD diagnosis during follow-up (adjusted analyses). |
| Hiltunen *et al*.  [54] (2013) | Kuopio 75+ cohort;  Finland | 5-year prospective, observational | 601 | Range 75-96 years | BNP at baseline | Diagnosis of dementia | Cross-sectional analyses: age, sex, medication, heart failure, and blood pressure. Prospective analyses: age, education, and hypertension. | Presence of any dementia associated with higher BNP in youngest tertile and lower BNP in oldest tertile.  Higher baseline BNP associated with increased risk of dementia during follow-up in youngest tertile only. |
| Tynkkynen *et al*. [56] (2015) | National FINRISK Study; Finland | 14-year prospective, observational | 7,158 | Mean 48 ± 13 years (range 25 to 74) | NT-proBNP at baseline | Medical records for diagnosis with any dementia or AD, or dementia medication | Age, sex, cholesterol, body mass index, ischemic heart disease, and diabetes | Higher baseline NT-proBNP associated with increased risk of any incident dementia across all participants. Finding driven by males. No finding on incident AD. |
| Vaes *et al*.  [55] (2009) | Leiden 85+ cohort;  The Netherlands | Cross-sectional, observational | 274 | All 90 years old | NT-proBNP categorized according to sex-specific tertiles of distribution | MMSE. ‘Poor cognitive function’ defined as MMSE <19. | Height, weight, renal function, hemoglobin, and cardiovascular medication | No association. |

ABI, ankle-brachial index; AD, Alzheimer’s disease; BNP, brain natriuretic peptide; cIMT, carotid intima-media thickness; FINRISK, Finland Cardiovascular Risk Study; MMSE, Mini-Mental State Examination; NT-proBNP, N-terminal pro-brain natriuretic peptide; VaD, vascular dementia.

{2 Column Table}

**Table S5. Studies of depression and cognitive function in type 2 diabetes**

| Study | Sample | Design | Number | Baseline mean age | Measurement of depression | Cognitive measures | Adjustment variables | Association with cognitive function |
| --- | --- | --- | --- | --- | --- | --- | --- | --- |
| Cukierman-Yaffe  *et al*. [13] (2009) | Patients with type 2 diabetes participating in ACCORD-MIND;  North America | Cross-sectional analysis of trial on blood pressure, lipids, and glycemic control | 2,977 | Mean 63 ± 6 years | Scores ≥10 on 9-item Patient Health Questionnaire or self-reported depression | Digit Symbol Coding (primary outcome), MMSE, Rey Auditory Verbal Learning, and Stroop (secondary outcomes) | Age | Association of depression with lower MMSE scores. No findings for other cognitive tests. |
| Katon *et al*. [60] (2012) | Patients with type 2 diabetes participating in the Diabetes and Aging Study;  USA | 3- to 5-year retrospective, observational | 19,000 | Range 30-75 years | ICD codes for diagnosis of depression/use of anti-depressants in hospital records | ICD codes for dementia in hospital records, restricted to incident cases occurring 3 to 5 years after baseline | Sociodemographic factors, clinical risk factors, health risk factors, and health use | Co-morbid depression/diabetes associated with increased risk of dementia diagnosis compared with diabetes alone. |
| Sullivan *et al*. [61] (2013) | Patients with type 2 diabetes participating in ACCORD-MIND;  North America | 40-month trial on blood pressure, lipids, and glycemic control | 2,977 | Mean age 63 ± 6 years | Scores ≥10 on 9-item Patient Health Questionnaire at baseline | Digit Symbol Coding (primary outcome), MMSE, Rey Auditory Verbal Learning, and Stroop (secondary outcomes) at baseline and 20 and 40 months | Age, sex, ethnicity, education, glycemia treatment arm, blood pressure versus lipid treatment arm, blood pressure treatment, lipid treatment, clinical center, cardiovascular event, body mass index, HbA1c, cholesterol, smoking, alcohol, and insulin use | Association of depression at baseline with steeper cognitive decline across cognitive tests. |
| Trento *et al*. [58] (2012) | Patients with 2 diabetes;  Italy | Cross-sectional, observational | 498 | Mean 68 ± 7 years | Zung Self-Rating Depression Questionnaire | MMSE | Age, sex, duration of diabetes, HbA1c, and insulin use | Statistically non-significant trend for association of higher depression scores with lower MMSE scores |
| Watari *et al*. [59] (2006) | Patients with type 2 diabetes;  USA | Cross-sectional, observational | 40 | Range 30-80 years. Mean 60 ± 12 years. | Clinical interview to diagnose depression | MMSE, 12 cognitive tests resulting in composite scores of attention/processing speed, memory, executive function, verbal memory, and non-verbal memory | Age, sex, and education | Statistically non-significant trend for lower overall cognitive function and statistically significant lower attention/processing speed in co-morbid depression/diabetes group than in no depression/diabetes group. |

ACCORD, Action to Control Cardiovascular Risk in Diabetes; ACCORD-MIND, ACCORD-MIND, Action to Control Cardiovascular Risk in Diabetes-Memory in Diabetes; ICD, International Classification of Diseases; MMSE, Mini-Mental State Examination.
